# The state prediction method of the silk dryer based on the GA-BP model

**DOI:** 10.1038/s41598-022-17714-x

**Published:** 2022-08-26

**Authors:** Hao Jiang, Zegang Yu, Yonghua Wang, Baowei Zhang, Jiuxiang Song, Jingdian Wei

**Affiliations:** School of Electrical Information Engineering, Zhengzhou University of Light Industry, Zhengzhou, 450000 People’s Republic of China

**Keywords:** Engineering, Mathematics and computing, Scientific data

## Abstract

Considering the under-maintenance and over-maintenance of existing equipment maintenance methods, this paper studies a Condition Based Maintenance method for silk dryers. The entropy method is used to eliminate the influence of subjective factors to more objectively reflect the weight of different input parameters; optimizing the number of nodes in the hidden layer of the network to improve the prediction accuracy; and using the GA-BP neural network to establish a state prediction model of the equipment to solve the disadvantages of the BP neural network, for example, unstable prediction, easily falling into local optimum, and slow global search ability. Simulation experiments show that this method can effectively compensate for the shortcomings of the existing maintenance methods, and provide an effective scientific basis for dryer state maintenance.

## Introduction

Modern industry continues to develop steadily, and an increasing number of machinery and equipment are being put into use. In order to avoid the influence of the failure of tobacco machinery and equipment^1^, reasonable maintenance is essential. In the tobacco production process, the maintenance methods of the dryer mainly include preventive maintenance and fault maintenance. Breakdown repair is an early form of equipment repair, which is the repair and maintenance of equipment after it has broken down. The type of maintenance is often accompanied by large economic losses and even casualties. Preventive maintenance is a hot topic in the field of equipment maintenance. It mainly includes cycle-based maintenance and Condition Based Maintenance. In addition, based on regular maintenance method, from the perspective of the application effect, due to the complex and unstable factors affecting the actual production process, even simple operations such as equipment lubricating oil supply and unit component inspection, there are still under-maintenance and over-maintenance. It is difficult to reach the expected effect. In contrast, the Condition Based Maintenance method is to take specific maintenance measures according to the different states of the equipment during operation, which is used to improve the effectiveness of preventive maintenance strategies^2^ and avoid insufficient and excessive maintenance.

Many scholars have made some progress in Condition Based Maintenance. Ye, et al. proposed to use weighted neural network to extract gearbox vibration signal features^3^. Tang, et al. proposed a fusion method of random forest mixture classifiers, which realized the diagnosis of a single fault in a complex fault environment^4^. CHRIS A presented a method for unsupervised process monitoring and fault diagnosis using machine learning methods^5^.

Based on state prediction, many scholars have also achieved some research results. The equipment state prediction methods mainly include artificial neural network modelling^6^, and deep learning prediction methods^7^, etc. The optimization algorithms mainly include genetic algorithm, simulated annealing algorithm, etc.

Traditional mapping function modelling cannot effectively remove the influence of noise, and cannot accurately simulate the change of the equipment state during the operation of the equipment. Scalabrini Sampaio Gustavo and others proposed a method for using artificial neural networks to predict the time of motor failure^8^. The artificial neural network that can predict the future condition of the equipment identities the time when the failure may occur. Sihan Chen and others proposed a wind turbine system fault early warning method based on a genetic algorithm to optimize Back Propagation (BP) neural network^9^. It is mainly based on the parameters monitored by the SCADA system and related analysis to realize the fault warning of the fan system. Luo Lincong and others proposed a medical equipment Condition Based Maintenance system based on BP neural network, which can predict the maintenance period, maintenance parts and maintenance items of medical equipment^10^. Zhihong Luo and others proposed a combined model of online condition monitoring technology based on SCADA data and BP neural network to predict equipment failures of wind turbines^11^. Qianjun Wu and others proposed a research and application method for a server health prediction model based on BP neural network^12^. In addition, the research of using the intelligent algorithm to predict parameters is developing gradually; particle swarm optimization (PSO)^13^, genetic algorithm (GA)^14^, neural network (NN), and other intelligent algorithms have been widely used in the prediction field. Yu et al. proposed a dynamic full-parameter adaptive BP neural network with genetic algorithm (GA) and BP neural network, and applied it to oil and gas reserve prediction^15^. Huang et al. proposed multi-feature fusion of the features obtained from the EMD method with some time-domain features as an input to the GA-BP algorithm to achieve fault diagnosis of bearings, which has a higher identification rate than using one feature^16^. Li et al. proposed a multi-sensor bearing fault diagnosis method based on GA-BP neural network^17^.

Based on the above research results, the application of BP neural networks is very extensive. BP neural networks have significant advantages in data fitting and function approximation^18^. The structure is simple and there are many training algorithms, but BP neural networks also have some defects, such as slow training speed, unstable prediction, ease of falling into local minima and slow global search ability^19^. Kajornrit J. proposed a method to optimize BP with a GA to improve the performance of BP neural networks^20^. D. N. Vishwakarma et al. proposed a GA-BP method for differential protection of power transformers^21^. Although the BP neural network trained by a GA can obtain more accurate results, but the results depend on the experience and knowledge of experts, and subjective factors have a greater impact.

In this paper, Genetic Algorithm (GA) is used to optimize the BP neural network, and it is applied to the state prediction of the silk drying machine. It can fit the mapping relationship between input samples and output samples well. In addition, because the distribution weight of the order relation method is affected by subjective arbitrariness, the result is completely dependent on expert experience and knowledge. To minimize the influence of subjective factors, this paper uses an entropy weight method based on objective data relations to redistribute the weight of input parameters. The method uses the entropy method to select input data, and can select reasonable data input samples; it avoids the influence of subjective factors, the BP neural network is optimized by a genetic algorithm, and a dryer state prediction model based on GA-BP neural network is constructed. Finally, the results are used for maintenance activities to make reasonable adjustments and dynamically adjust equipment maintenance plans based on equipment operating data to ensure equipment reliability while avoiding excessive maintenance and improving equipment maintenance efficiency.

The first part of the article introduces the structure and research content of the article, the second part introduces the entropy weight analysis method and the GA-BP model, and the third part introduces the initial data and brings the data into the model. After the data comparison chart is obtained in the fourth part, the GA-BP and BP data are compared. The fifth part concludes that the GA-BP model improves the average prediction accuracy, lays a foundation for equipment maintenance, and improves maintenance efficiency.

## Model building

### Entropy weight analysis method

The entropy weight method is based on the degree of variation in feature parameters, and obtains the entropy weight of each feature parameter through information entropy, and then obtains the weight of each feature parameter^22^. The greater the difference of an indicator, the smaller the entropy weight, which indicates that the greater the amount of information provided by the indicator, the greater the role it plays in the evaluation, and the greater the weight, which provides a basis for comprehensive evaluation. The calculation process of the entropy weight of the input parameter is as follows: calculate the entropy value under this output parameter according to formula (1):

1 $$ e_{j} = - \frac{1}{\ln n}y_{ij}^{^{\prime}} \mathop \sum \limits_{i = 1}^{m} y_{ij}^{^{\prime}} $$

In formula (1),${ }y_{ij}^{^{\prime}} = \frac{{y_{ij} }}{{\mathop \sum \nolimits_{i = 1}^{m} y_{ij} }}$,is the proportion of the *i* sample data under the *j* characteristic parameter, ${\text{y}}_{ij}$ is sample data, *m* is the number of sample data, *n* is the number of feature parameters;

According to the entropy value of each index calculated according to formula (1), their weight is calculated according to formula (2):

2 $$ q_{j} = \frac{{1 - e_{j} }}{{n - \mathop \sum \nolimits_{j = 1}^{n} e_{j} }} $$

In formula (2), *n* is the number of characteristic parameters; ${\text{e}}_{j}$ is the entropy weight of the *j* feature parameter; $q_{j}$ is the weight value of the j feature parameter obtained by the entropy method.

### GA-BP algorithm model

**Figure 1:**
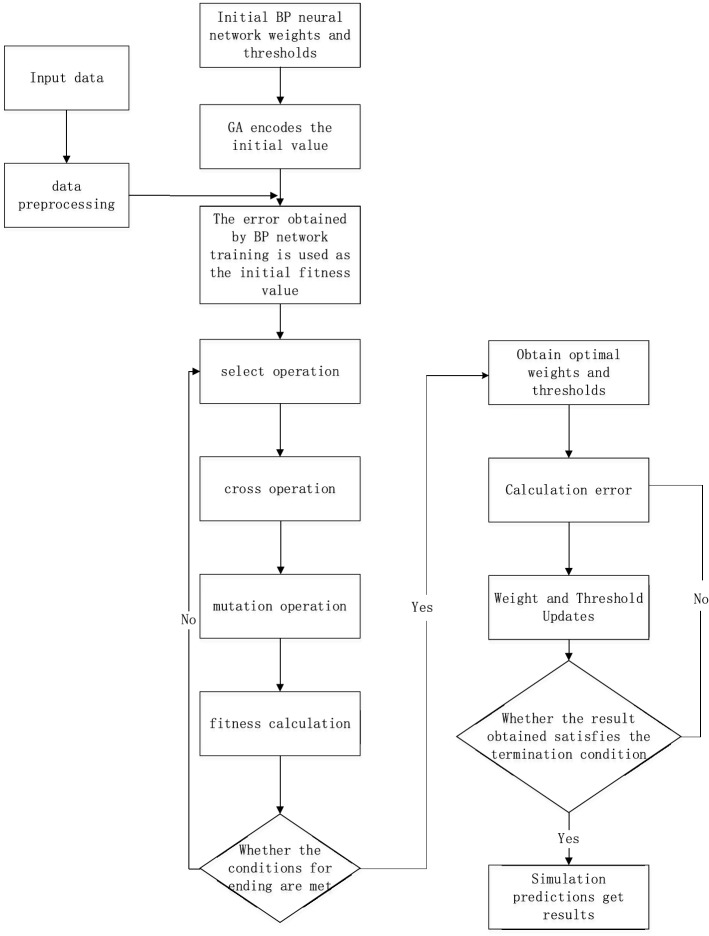
GA-BP neural network flow chart.

The genetic algorithm (GA) has strong macro search and global optimization capabilities, which can solve the local minimal problems of the network and improve the network performance. Currently, the GA is commonly used to optimize the BP neural network^23^. The GA-BP algorithm flow chart is shown in Fig. 1, and the specific steps are as follows.

1. Population initialization

Determine the population number, evolution times, population size, crossover probability, and mutation probability, and build the initial population.

(2) Initialize the BP network model

Set the input layer node, output layer node, and hidden layer node of the neural network according to the training set data, where the hidden layer node is obtained by the empirical formula (3).

3 $$ {\text{L}} = \sqrt {m + n} + l $$

In the formula, m is the number of input layer nodes, n is the number of output layer nodes, which is an arbitrary constant between^1,10^.

(3) Determine fitness function

Fitness refers to the degree to which each individual in the measurement group is close to the optimal solution in the optimization calculation, and predicts the absolute value of the error between the expected outputs and the calculation formula for the individual fitness value as formula (4):

4 $$ {\text{F}} = {\text{k}}\left( {\mathop \sum \limits_{i = 1}^{n} abs\left( {y_{i} - o_{i} } \right)} \right) $$

In the formula, *n* is the number of training samples, $$y_{i}$$ is the actual value of the *i* node of the BP neural network, $$ o_{i}$$ is the predicted output of the *i* node; $${\text{k}}$$ is the coefficient, generally $$\frac{1}{n}$$.

(4) Select operation

The roulette method is used to select the chromosome with the highest fitness calculated by formula (2) from the current population for the copy operation, and this process generates a new population. Among them, the probability of selecting each individual is calculated by formula (5):

5 $$ p_{i} = {\text{F}}\left( {x_{i} } \right)/\mathop \sum \limits_{1}^{n} F\left( {x_{i} } \right) $$

In the formula, *n* is the number of individual populations; Is the individual $x_{i}$ fitness value calculated by formula (4).

(5) Cross operation

Using the real number crossover method, the crossover operation between the ${\text{k}}$ chromosome $a_{k}$ and the *l* chromosome $a_{j}$ at position *j* is shown in formula (6):

6 $$ \left\{ {\begin{array}{*{20}c} {a_{kj} = a_{kj} \left( {1 - b} \right) + a_{lj} b} \\ {a_{lj} = a_{lj} \left( {1 - b} \right) + a_{kj} b} \\ \end{array} } \right. $$

In this formula includes a random number and ${\text{b}} \in \left[ {0,1} \right]$.

(6) Mutation operation

Selecting the *j* gene of the *i* individual to mutate to obtain a brand-new individual, the method is shown in formula (7):

7 $$ \left\{ {\begin{array}{*{20}c} {a_{ij} = a_{ij} + \left( {a_{ij} - a_{\max } } \right) \times f\left( g \right) ,r > 0.5} \\ {a_{ij} = a_{ij} + \left( {a_{\min } - a_{ij} } \right) \times f\left( g \right) ,r \le 0.5} \\ \end{array} } \right. $$

In this formula, $$a_{\max }$$ and $$a_{\min }$$ are the upper and lower bounds of gene $$a_{ij}$$, $$ f\left( g \right) = r_{2} \left[ {\left( {1 - g} \right)/G_{\max } } \right]^{2}$$, $$ r_{2}$$ is a random number, $${\text{g}}$$ is evolutionary algebra, $$G_{\max }$$ is the maximum number of evolutions; $$r $$ is a random number and $$ r \in \left( {0,1} \right)$$.

(7) Calculate fitness

Replace the original chromosome with the new chromosome to calculate the new individual fitness. If the fitness meets the condition, skip to step (8); otherwise, go to step (3) to continue calculating the fitness.

(8) Train

After the performance indicators are reached, the optimal weights and thresholds are assigned to the BP neural network, and the training set is used to train the network until the set error requirements are reached.

## Model settings

### Data sample

According to silk dryer maintenance plans, the data samples of fixed period intervals after each lubrication maintenance are read from the equipment parameter library, and the collected data samples are divided into three parts: process parameters, state parameters and electrical parameters. Process parameters include the actual moisture at the drying wire outlet, drying wire outlet temperature, ambient temperature, and ambient humidity. State parameters include the motor vibration state and equipment lubrication state. Electrical parameters include the three-phase current, three-phase active power, three-phase reactive power, power factor, frequency, active energy, and reactive energy. The above data samples are closely related to the maintenance of the silk dryer. During its fixed period, the dryer lubrication state is the theoretically calculated value. Since the current equipment maintenance adopts a cycle-based maintenance method, the lubrication state of the equipment can be regarded as a descending process of the Weibull distribution^24^. After the silk dryer is lubricated and maintained, the lubricating state is the initial characteristic value of 10, that is, the equipment is well lubricated, and no lubricant needs to be added. Before the next lubrication maintenance, the lubrication state of the equipment is taken as the critical characteristic value 1, which means that the lubrication state of the equipment is poor, and it is necessary to stop the machine to add lubricant.

In this paper, GA-BP is used to predict the state of the equipment. To improve the accuracy and universality of state prediction, this paper divides the 100 collected sets of data, selects the first 80 sets of data as the training set of the prediction model, and selects the last 20 sets of data as the test set. The raw data have different dimensions, and the range of values varies widely. To ensure the training speed of the network and facilitate the calculation, it is necessary to normalize the raw data collected in the database. This paper uses max–min normalization to linearly transform the original data into the range [0,1]. The normalized transformation formula is shown in Eq. (8). After the experimental results are obtained, inverse normalization is performed to reduce the normalized value to the true value.

8 $$ x^{*} = \left( {x - x_{\min } } \right)/\left( {x_{\max } - x_{\min } } \right) $$

In the formula $$ x^{*} { }$$ represents the normalized value of the data, $$x$$ is the original value of the data, $$x_{\min }$$ is the minimum value of the sample data, $$x_{\max }$$ is the maximum value of the sample data.

Because the relationship between the state parameters, process parameters and electrical parameters of the equipment is complex, and there are ambiguities and uncertainties, the electrical parameters and process parameters of the equipment are used as input parameters, and the state parameters are used as output parameters. In order to analyze the influence of the input parameters on the output parameters, the entropy weight method is used to determine the correlation degree and weight order of each parameter to the output parameters. The results are shown in Tables 1 and 2.

**Table 1:** Entropy weights of various factors on motor vibration acceleration.

Correlation	q	Order
Actual moisture	0.857	1
output temperature	0.835	2
Ambient temperature	0.723	4
environment humidity	0.572	7
Three-phase current	0.482	9
Active power	0.668	5
Reactive power	0.623	6
Power factor	0.813	3
Active energy	0.451	10
Reactive energy	0.430	11
frequency	0.489	8

**Table 2:** Entropy weights of various factors on equipment lubrication status.

Correlation	q	Order
Actual moisture	0.887	1
output temperature	0.821	2
Ambient temperature	0.792	3
environment humidity	0.759	4
Three-phase current	0.463	8
Active power	0.621	6
Reactive power	0.582	7
Power factor	0.698	5
Active energy	0.437	10
Reactive energy	0.414	11
frequency	0.428	9

Table 1 shows that the order of the degree of influence on the vibration state of the motor is actual moisture at the outlet of the drying wire > temperature of the outlet of the drying wire > power factor > ambient temperature > three-phase active power > three-phase reactive power > environmental humidity > frequency > three-phase current > active power > reactive power;

Table 2 shows that the order of the degree of influence on the lubrication status of the equipment is the actual moisture at the outlet of the drying wire > the temperature of the outlet of the drying wire > the ambient temperature > the ambient humidity > the power factor > the three-phase active power > the three-phase reactive power > the three-phase current > the frequency > active energy > reactive energy. Taking into account the closeness of the correlation between input and output, the final selection of drying wire outlet temperature, drying wire outlet actual moisture, power factor, ambient temperature, three-phase active power, three-phase reactive power and environmental humidity are used as the input layer parameters of the neural network, to streamline the predictive neural network model structure.

### Parameter settings

**Figure 2:**
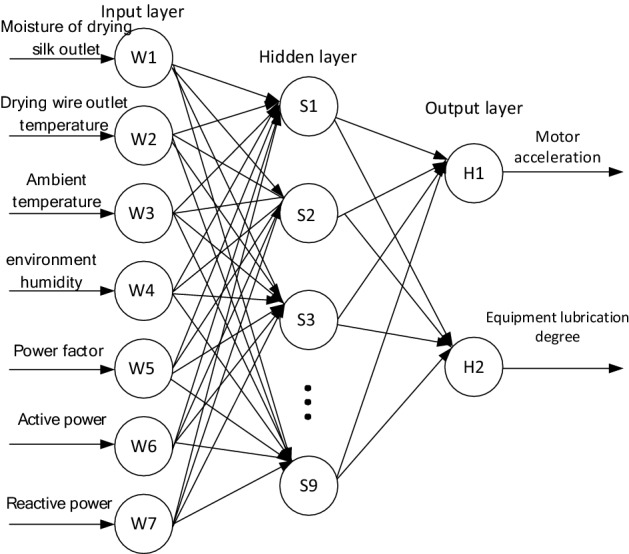
BP neural network model structure.

This paper uses a three-layer neural network, and the layers are fully connected, and the same layers are not connected. Set the number of neurons in the input layer of the neural network to 7 and the number of neurons in the output layer to 2. The neural network topology is shown in Fig. 2. Therefore, according to the hidden layer empirical formula (3), the number of hidden layer nodes is calculated as an arbitrary integer between^3,13^. In order to optimize the structure of the neural network and ensure the best prediction intensive reading after training, when building the neural network, the different odd numbers in 3–13 are substituted in sequence and set as the number of hidden layer nodes, and the models under different schemes are compared to reach the target ratio and running time. To obtain get the proper number of hidden layer nodes of the GA-BP neural network. The comparison results of different hidden layer neuron number schemes are shown in Table 3, and the algorithm parameter settings are shown in Table 4.

**Table 3:** Comparison of the number of neurons in different hidden layers.

Hidden layer neuron	Proportion reached /%	Running time/s
3	62	3.53
5	71	3.25
7	79	3.12
9	90	2.85
11	87	2.82
13	82	2.78

**Table 4:** Algorithm parameter settings.

Training times	Number of input layers	Number of hidden layers	Number of output layers	
1000	7	9	2	

Activation function	Training function	Learning rate	Training goal	
ReLU	LM	0.01	0.0001	

Total group number	Maximum genetic algebra	Crossover probability	Mutation probability	Generation gap GAP
50	500	0.8	0.05	0.9

As seen in Table 3, when the number of neurons in the hidden layer is 3 ~ 13, with the increase in the number of neurons in the hidden layer, the proportion reaching the training target shows a trend of first increasing and then decreasing, and the running time of the prediction model is predicted. It is negatively correlated with the number of neurons in the hidden layer. In order to ensure the convergence speed of the overall model and the stability of the prediction, the final number of hidden layers is set to 9.

## Experimental results

### Algorithm comparison

**Figure 3:**
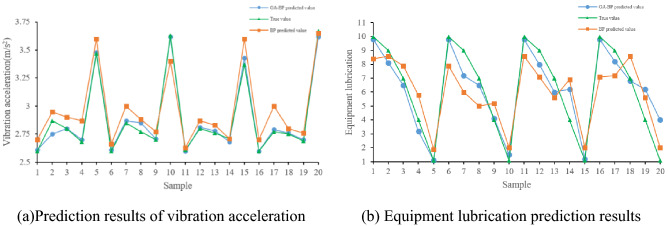
Forecast results.

**Figure 4:**
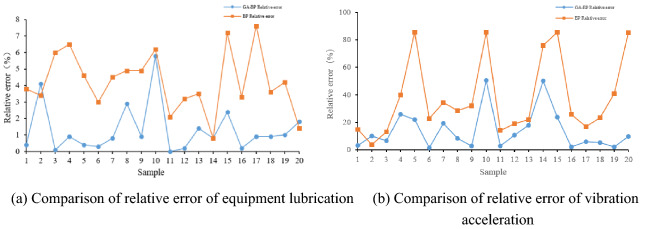
Prediction error.

In order to verify the predictive performance of the model, a BP neural network model and a GA-BP neural network model are constructed using the Python platform framework. The same training samples and test samples are used to train and predict two different neural network models, and then the different prediction results are compared. Figure 3 shows the prediction results of the BP model and the GA-BP model, and Fig. 4 shows the relative error comparison of the two models. Table 5 compares the relative errors of the two models under the motor vibration acceleration. Table 6 compares the relative errors of the two models under the same equipment lubrication conditions.

**Table 5:** Prediction error of motor vibration acceleration/%.

Vibration acceleration	01	02	03	04	05	06	07	08	09	10
GA-BP	0.4	4.1	0.1	0.9	0.4	0.3	0.8	2.9	0.9	5.8
BP	3.8	3.4	6.0	6.5	4.6	3.0	4.5	4.9	4.9	6.2

Vibration acceleration	11	12	13	14	15	16	17	18	19	20
GA-BP	0	0.2	1.4	0.8	2.4	0.2	0.9	0.9	1.0	1.8
BP	2.1	3.2	3.5	0.8	7.2	3.3	7.6	3.6	4.2	1.4

**Table 6:** Prediction error of equipment lubrication degree/%.

Lubrication status	01	02	03	04	05	06	07	08	09	10
GA-BP	3.3	10.1	6.5	26.0	22.1	1.5	19.4	8.5	2.7	50.5
BP	15.0	3.8	13.3	39.9	85.4	22.6	34.4	28.6	32.1	85.5

Lubrication status	11	12	13	14	15	16	17	18	19	20
GA-BP	2.8	10.9	17.9	50.3	23.9	2.1	5.9	5.4	2.3	9.6
BP	14.2	18.9	21.9	75.8	85.4	26.0	17.0	23.5	40.8	85.2

The experimental results show that both models can predict the output to a certain extent, but the GA-BP algorithm model has a significant improvement in prediction accuracy compared with the traditional BP neural network model, mainly because the BP neural network uses the gradient search algorithm constraint, which easily falls into the local optimum, so the prediction error of the lubrication state of some equipment is very large. The GA-BP neural network model partially optimizes the initial weights and thresholds of the network due to the genetic algorithm, which significantly improves the prediction accuracy. Among them, in the prediction of the motor vibration acceleration by the BP neural network algorithm model and the GA-BP neural network algorithm model, the maximum relative errors are 7.6% and 5.8%, the minimum relative errors are 0.8% and 0.0%, and the average relative error values are 4.24% and 1.31%. In the prediction of equipment lubrication status by the BP neural network algorithm model and GA-BP neural network algorithm model, the maximum relative error is 85.5% and 50.5%, the minimum relative error is 3.8% and 1.5%, and the average relative error is 38.47% and 14.09%, respectively. The GA-BP neural network algorithm has a significantly better prediction accuracy than the BP neural network algorithm. However, this prediction model still has some shortcomings. For example, the parameter selection in the GA algorithm will seriously affect the prediction accuracy and model training, and the fitness iteration time is longer.

### Forecast application

This method adopts two output quantities of motor vibration acceleration and equipment lubrication degree, and adopts different maintenance measures for the silk dryer in different equipment states to solve the problem of improper maintenance in current equipment maintenance. According to historical data and expert experience, when the predicted value of motor vibration acceleration is higher than 3.7 m/s^2^, professional operators will perform the shutdown maintenance of the motor unit; when the vibration acceleration is between 2.8 and 3.7 m/s^2^, equipment operating parameters and component status focus are performed on observation and determine the corresponding motor maintenance strategy; when the vibration acceleration is lower than 2.8 m/s^2^, the equipment is considered to be operating normally and requires no maintenance. The lubrication status parameter prediction of the equipment is related to the amount of lubricating grease injected. The larger the predicted value, the better the lubrication degree of the equipment, and the lower the injected amount of lubricating grease. According to different predicted values as a reference for the lubrication status of the equipment, a reasonable amount of lubricating oil and grease injected is selected to achieve the purpose of preventive maintenance. The proposed method can reduce the maintenance cost of equipment, and can provide an effective reference for the transition from regular maintenance to state maintenance.

## Conclusion

Aiming at the problems of under-maintenance and over-maintenance in the current equipment maintenance process, an equipment state prediction method based on electrical parameters and process parameters was proposed. In this paper, the entropy method is used to analyze the main factors affecting equipment maintenance, which avoids the influence of subjective factors; the neural network is used to predict the equipment status, the number of input layer nodes of the neural network is optimized, and the network structure is simplified. The improved GA-BP model reduces the average error of the equipment lubrication state from 38.47% to 14.09%, which improves the accuracy of the average prediction. Further, the GA-BP neural network model is applied to the state prediction of the drying machine, which lays a foundation for equipment maintenance and improves maintenance efficiency. Additionally, the dryer state prediction reduces the consumption of manpower and material resources, reduces equipment accidents and personal accidents, and improves equipment operation stability.

## Data Availability

The datasets generated during the current study is not public as it is an actual project of the company and is confidential to the company, but are available upon reasonable request of the corresponding author.
